# Sex Differences in Cardiac and Clinical Phenotypes and Their Relation to Outcomes in Patients with Heart Failure

**DOI:** 10.3390/jpm14020201

**Published:** 2024-02-12

**Authors:** Akane Kawai, Yuji Nagatomo, Midori Yukino-Iwashita, Ryota Nakazawa, Yusuke Yumita, Akira Taruoka, Asako Takefuji, Risako Yasuda, Takumi Toya, Yukinori Ikegami, Nobuyuki Masaki, Takeshi Adachi

**Affiliations:** 1 Department of Cardiology, National Defense Medical College, Tokorozawa 359-8513, Japan; 2 Department of Intensive Care, National Defense Medical College, Tokorozawa 359-8513, Japan

**Keywords:** sex difference, heart failure, supra-normal EF, guideline-directed medical therapy

## Abstract

Biological sex is one of the major factors characterizing the heart failure (HF) patient phenotype. Understanding sex-related differences in HF is crucial to implement personalized care for HF patients with various phenotypes. There are sex differences in left ventricular (LV) remodeling patterns in the HF setting, namely, more likely concentric remodeling and diastolic dysfunction in women and eccentric remodeling and systolic dysfunction in men. Recently supra-normal EF (snLVEF) has been recognized as a risk of worse outcome. This pathology might be more relevant in female patients. The possible mechanism may be through coronary microvascular dysfunction and sympathetic nerve overactivation from the findings of previous studies. Further, estrogen deficit might play a significant role in this pathophysiology. The sex difference in body composition may also be related to the difference in LV remodeling and outcome. Lower implementation in guideline-directed medical therapy (GDMT) in female HFrEF patients might also be one of the factors related to sex differences in relation to outcomes. In this review, we will discuss the sex differences in cardiac and clinical phenotypes and their relation to outcomes in HF patients and further discuss how to provide appropriate treatment strategies for female patients.

## 1. Introduction

Heart failure (HF) is a leading cause of death and a major socioeconomic problem that has been increasing in prevalence worldwide [1]. Nonetheless, the prognosis of HF remains poor. On the other hand, recent advances in novel agents and modalities for HF allow us to provide alternative treatments, such as pharmacological therapy, device therapy, mechanical circulatory support, or heart transplantation. Particularly, the progress of pharmacological therapy has been remarkable over the past several decades [2,3,4]. β-blocker, renin–angiotensin system inhibitor (RASi), and mineralocorticoid receptor antagonist (MRA) have been reported to improve the prognosis of HF patients. In addition to these agents, recent evidence suggests that angiotensin receptor neprilysin inhibitor (ARNI) and sodium-glucose cotransporter 2 inhibitor (SGLT2i) are efficacious in terms of improved cardiac function, quality of life, and prognosis of HF, and they have been recognized as the standard medication for HF treatment [5,6]. Ivabradine and vericiguat, which have a unique mechanism of action, are also indicated to treat HF [7,8]. HF patients have various etiologies and show diverse phenotypes. Over the last two decades, HF with preserved ejection fraction (HFpEF), which is primarily caused by diastolic dysfunction of the left ventricle (LV), has emerged and has been recognized as a novel HF phenotype [9]. Cancer therapy-related cardiac dysfunction has also emerged along with recent advances in cancer therapeutics and improved patient prognosis for those with advanced cancer [10]. In this context, we need to choose the most suitable treatment and management, not in a one-size-fits-all approach but in a personalized manner.

In the United States, the age-adjusted prevalence of HF is lower in females than males. However, among individuals > 70 years of age, the absolute number of individuals with HF is higher in females, and this age group doubled the absolute count between 1990 and 2019 [11]. Biological sex is one of the major factors characterizing HF patients’ phenotype, and a variety of sex-related differences among HF patients have been reported [12,13,14]. Therefore, understanding sex-related differences in HF is crucial to implement personalized care for HF patients with various phenotypes. Hence, in this review, we summarize the current knowledge of the sex-related differences in cardiac and clinical phenotypes, medical treatment, and clinical outcomes in HF. Further, we discuss the potential mechanisms of sex-related differences in HF pathophysiology.

## 2. Sex Differences in Heart Morphology and Structure

In healthy populations, older patients have smaller LV chamber sizes and higher LV ejection fraction (LVEF) in both sexes [15]. The LV size is smaller in women than men throughout life. Interestingly, in patients > 70 years old, an increase in LVEF was observed only in females [16]. In autopsy samples of ventricular tissues from human hearts, the number of myocytes decreased with age in men but not in women [17]. This result suggests that LV systolic function might be preserved with age in women compared to men. On the other hand, the fibrosis in the extracellular matrix of myocardial tissue was more advanced in women than men among the elderly [18] and even in a healthy population. Female sex was associated with greater age-dependent increases in LV stiffness compared to males [16].

Sex-related differences in LV remodeling with hypertension were also observed, with more concentric hypertrophy and higher LVEF noted in women and more eccentric remodeling observed in men [19,20,21]. Similarly, in the HFpEF population, women were reported to have more prevalent concentric remodeling or hypertrophy and higher LVEF, although its mechanism has not been fully elucidated [22]. Smaller LV chamber sizes in women may lead to the hypercontraction of LV, and this may, at least in part, explain this phenomenon [22]. According to the guidelines, the cutoff of LVEF, which defines the categories of HF, is identical between men and women (e.g., LVEF ≥ 50% for HFpEF). However, despite higher LVEF in women, the global LV strain was similar between the sexes, and the mitral valve S’ velocity was somewhat lower in women, suggesting that the systolic function in women was not favorable compared to men [22]. It was also reported that, for a given LVEF, women have subclinical evidence of contractile dysfunction, such as reduced systolic twist and apical rotation [23]. For example, LVEF of 50% in a woman with HF may reflect a relatively lower systolic function compared to a man with the same LVEF. This hypothesis might explain the mechanism of greater benefit from agents such as sacubitril/valsartan and mineralocorticoid receptor antagonists in women for a higher range of LVEF compared to men [24]. 

A preclinical study suggested that a smaller LV size was related to more enhanced stiffness of the LV [25]. The small LV, the stiffness of LV, and higher LVEF (=supra-normal LVEF: snLVEF) could be closely related to each other. And this phenomenon was more frequently observed in women. In HFpEF, female patients showed more impaired diastolic function such as lower e’ and higher E/e’ than male patients [22]. Both LV diastolic and systolic stiffness were higher in women than men, even when adjusting for LV concentricity and clinical covariates [22]. Another study reported that Ea (arterial elastance) and Ees (end-systolic elastance) were both higher in women than men, indicating more advanced vascular and ventricular stiffness in HFpEF [22]. Women with HFpEF had higher pulmonary capillary wedge pressure (PCWP), both at rest and peak exercise, higher LV filling pressure, and lower increment in stroke volume index (SVi) with exercise [26]. These might be related to lower exercise capacity in women [26]. 

## 3. The Sex Differences in Cardiac Remodeling Related to Body Composition in HF 

Among a variety of risk factors for HF, obesity is an important risk factor, which shows sex differences in HF. Among postmenopausal women, obesity was the second-highest risk factor for HFpEF following hypertension [27]. Particularly, the presence of central obesity, not general obesity, was reported to be related to depressed LV systolic and diastolic function [28,29]. Interestingly, whereas higher body mass index (BMI) (but not waist circumference (WC) and waist–hip ratio (WHR)) was associated with higher LVEF, higher WC and higher WHR (but not BMI) were associated with lower global longitudinal strain [29]. This type of obesity is more frequently observed in women after menopause. The increase in visceral adipose tissue after menopause is reported to be correlated with an increase in the testosterone concentration rather than a decrease in estradiol [30]. The impact of obesity on incident HF was reported to differ in men and women. Although higher BMI portended a higher risk of HFpEF compared with HFrEF, the differential association of BMI with HFpEF versus HFrEF was more pronounced among women when compared with men [31]. Several possible mechanisms, such as insulin resistance, systemic inflammation, hypertension, and microvascular dysfunction, were suggested in previous research findings [31], but they have not been fully elucidated. On the other hand, in HFrEF patients, the impact of obesity on incident HF was higher in men than women [31]. This differential impact of obesity on HF subtypes by sexes might be key to understanding the mechanism of sex-related differences in HF phenotypes. 

Female HF patients have lower BMI [32], which is also associated with poor prognosis, often referred to as the obesity paradox [32]. Lower BMI is especially noted in Asian populations, including those with HF [32,33]. Lower body weight is associated with frailty and/or sarcopenia. Frailty is classically defined as the presence of the following criteria: unintentional weight loss, slow gait speed, weak grip strength, physical exhaustion, or low physical activity [34]. However, the concept of frailty has been broadened and is now defined as a deterioration of the multidimensional and multisystem condition, characterized by decreased functional reserves and increased vulnerability to stress and acute adverse events, which includes physical, cognitive, or social impairments [35]. The high prevalence of frailty, particularly in patients with HF [36] and its association with poor outcomes [37,38], is well established. Systematic inflammation might be one of the possible mechanisms mediating deteriorated HF pathophysiology based on previous research findings [39,40,41]. Over the past 2 decades, the higher prevalence of frailty in women versus men has been consistently reported in large, community-dwelling populations [38,42,43] and more recently in HF patients [44,45]. This may be related to a worse quality of life (QOL) in female HF patients [46,47]. 

Sarcopenia and frailty sometimes retrieve a similar clinical picture, but these two terms differ substantially in terms of their concept. Sarcopenia is a syndrome characterized by progressive and generalized loss of skeletal muscle mass and strength with a risk of adverse outcomes, such as physical disability, poor quality of life, and clinical outcome [48,49,50,51]. In the previously conducted studies, including our group [52,53], female sex was independently associated with lower psoas muscle mass, measured by computed tomography. Also, we found E/e’, an index of diastolic function, was negatively associated with psoas muscle mass [52], which was in line with previous reports that showed negative correlations of E/e’ with skeletal muscle mass [54,55]. Interestingly, this association was observed only in women and not in men [52]. As a potential mechanism, elevated LV end-diastolic pressure can cause pulmonary congestion accompanied by oxygen desaturation on exertion or even at rest, which can eventually lead to decreased physical activity. A sequence of these physiological responses may result in disuse muscle atrophy in elderly patients with HF. On the other hand, lower muscle mass is related to insulin resistance [56], which can cause the exacerbation of diastolic dysfunction [54]. Collectively, LV diastolic dysfunction can potentially lead to decreased muscle mass and vice versa. Their causal relationship and detailed mechanisms remain unproved and need further investigation.

Recently, the impacts of physical frailty on the outcomes of HF patients were reported to be different between men and women. In contrast, due to the higher prevalence of physical frailty and lower QOL among female HF patients, the outcomes of HF were reported be more favorable in frail HF women than frail HF men [57,58]. The cause of this paradox is totally unknown and needs to be explored, since it is important to better understand sex differences in the outcomes of the HF population.

## 4. Supra-Normal EF

Recently, a large regional healthcare-system-based study reported that adjusted hazard ratios for mortality showed a U-shaped relationship for LVEF, with a nadir of risk at an LVEF of 60% to 65%. Although this relationship was observed in both sexes in all age-stratified groups, it was more evident in female patients, with a significant interaction between sexes [59]. In another study, enrolling subjects with EF ≥ 57%, higher LVEF was significantly associated with an increased risk of major adverse cardiovascular events (MACE) among individuals with low but not high stroke volume [60]. Thus, higher LVEF, often referred to as snLVEF, has been identified as a risk of adverse cardiac events. 

An association between snEF and increased mortality might be particularly relevant in the female population, whereas men do not show the same relationship. Other studies enrolling patients who underwent non-invasive imaging modality testing indicate that women with snEF had a higher risk of mortality [61,62] and MACE [63], whereas men did not show the same relationship. 

**Figure 1:**
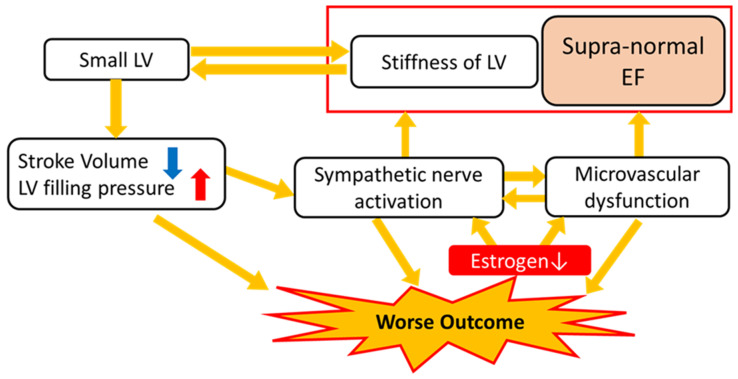
The proposed mechanisms mediating worse outcome by snEF in women. The snEF might reflect LV stiffening rather than enhanced systolic function (see the Discussion). Small LV size was also shown to be associated with LV stiffness. Small LV size can cause lower cardiac output and elevated LV filling pressure, which can lead to sympathetic nerve activation. Microvascular dysfunction and sympathetic nerve activation are shown to be associated with snEF, exclusively in women. Since estrogen is shown to attenuate sympathetic nervous tone and favorably regulate coronary microcirculation, especially women after menopause might be more susceptible to these pathologies. These could explain the worse outcomes of female patients with snEF. LVEF, left ventricular ejection fraction; snEF, supra-normal left ventricular ejection fraction.

The potential mechanism mediating snEF and increased mortality in female patients remains unknown. One possible mechanism is coronary microvascular dysfunction. Coronary flow reserve (CFR), which is an indicator of coronary microvascular disease, was reported to be related to E/e’, an indicator of the LV filling pressure in patients with type-2 diabetes, even after the adjustment for covariates [64]. A previous study examined the parameter of echocardiography in snLVEF patients, consisting of 80% women and compared to normal LVEF. This study findings showed that the morphology of hearts with snLVEF showed lower RWT and smaller LV volume compared to those with normal LVEF. Further, the hemodynamic analysis showed increased Ees and Eed, which suggested increased LV stiffness, both at the systole and diastole [65]. According to a previous study using ^13^N-ammonia positron emission tomography, women with snEF showed reduced coronary flow reserve and blunted heart rate response to adenosine infusion, indicating microvascular dysfunction and heightened sympathetic nerve activity. This association was not observed in men [63]. As LV hypercontractility and cardiac sympathetic hyperactivity have been observed in patients with coronary microvascular dysfunction [66], these features could reflect the mechanism underlying the poorer prognosis in this population (Figure 1). Sex hormones have been considered as potential candidates mediating this pathophysiology. Previous studies suggested that estrogen could attenuate sympathetic nervous tone in humans [67] and can also favorably regulate coronary microcirculation through the production of endothelial nitric oxide synthase [68,69]. Moreover, a lack of testosterone might cause impaired myocardial perfusion, which has been demonstrated in mice [70]. Therefore, women especially after menopause might be more susceptible to these pathologies through a sex hormone deficit, leading to worse outcomes accompanied by snEF.

Taken together, worsened LV stiffness, smaller LV volume, microvascular dysfunction, and sympathetic nerve overactivation might, at least in part, account for the increase in cardiac events observed in women with snEF (Figure 1).

## 5. The Sex-Related Differences in Guideline-Directed Medical Therapy (GDMT)

In clinical practice, the quality of medical care has also been reported to differ between male and female patients. GDMT has been a cornerstone of the treatment for HFrEF according to accumulating evidence in the last three decades [2,3,5,8,71,72]. Several studies reported that female HFrEF patients were associated with a lower implementation of GDMT (Table 1) [73]. Particularly, the prescription rates of β-blocker and RASis were significantly lower in women than men. Notably, our group reported that a lower prescription rate of these agents in women was observed in HF with mildly reduced EF (HFmrEF, 40% ≤ LVEF < 50%) but not in HFrEF (LVEF < 40%) [74]. Compared to these, the prescription rate of MRA did not largely differ, and, rather, one study reported that it was higher in women than men [14]. This may be due to MRA’s distinctive sex-hormone-related side effects, such as gynecomastia or reduced fertility, leading to withholding or a cessation of MRA treatment in male patients [3]. The data on sex-related differences in the prescription rate of SGLT2i and ARNI are not yet sufficient, and further investigation is needed.

The reason for a lower prescription of GDMT in women is unclear. One of the possible factors might be adverse reactions to medicines. The emergence of adverse drug reactions (ADRs) could lead to a discontinuation of medical therapy. ADRs were reported to be more frequent among women than men at all ages, and the symptoms of ADR were more highly divergent in women [84,85]. Moreover, the discontinuation of drugs due to ADRs was more frequent in women than men [86,87]. One of the possible mechanisms which can explain that women are more likely to experience ADRs than men could be the sex-related differences in pharmacokinetics. The representative β-blockers, metoprolol and propranolol, which are metabolized through CYP2D6, are reported to show a higher plasma level in women than men, because men have greater activity of CYP2D6 than women. Testosterone can induce the expression of this enzyme [88]. Some ACEis or ARBs are reported to show a higher blood concentration in female patients than male patients because of their lower body weight [89]. From these mechanisms, female patients more often tend to face side effects from GDMT. It might be necessary to determine the accurate dose of GDMT for men and women [90]. As for the other potential factors related to a lower implementation of GDMT in women, a higher prevalence of physical frailty [44] and comorbidities [43] including depression [91] or lower social support was reported in women compared to men [92,93]. Further, in women, the prescription might be more likely to be declined and, thus, less likely to be recommended by the attending physician [87].

As a novel agent, SGLT2i use was also reported to be lower in women with both HF and DM [94,95]. It is possible that the attending physicians may hesitate to prescribe SGLT2i because of concerns over increasing the risk of urinary tract infections, especially among elderly women [96,97]. In patient-level pooled analysis of DAPA-HF (Dapagliflozin and Prevention of Adverse Outcomes in Heart Failure) and DELIVER (Dapagliflozin Evaluation to Improve the Lives of Patients with Preserved Ejection Fraction Heart Failure), dapagliflozin reduced the primary endpoint, defined as the composite of cardiovascular death and worsening HF events in both men and women similarly, with no sex-related differences or safety events. The benefit of dapagliflozin was observed across the entire ejection fraction spectrum and was not modified by sex. There were no sex-related differences in serious adverse events, adverse events, or drug discontinuation attributable to adverse events [98]. From these findings, SGLT-2i might need to be more prescribed in women. ARNI is also reported to be less prescribed in women [82]. In the PARAGON-HF trial, in a study of a population that included HF with LVEF of 45% or greater, the ARNI group showed a significantly lower incidence of the primary endpoint compared to a placebo group only in women [99]. As for the potential mechanism of this sex difference, the following has been discussed. Women in this study were more obese, and obesity is related to insufficient BNP secretion; ARNI may be more effective in inhibiting the degradation of BNP. And, from the findings of PARADIGM-HF, which enrolled HFrEF patients and showed significant a cardiovascular event lowering effect [5], ARNI might be more effective for systolic dysfunction. As discussed above, a given LVEF in a woman may reflect a relatively lower systolic function compared to a man with the same LVEF. So, from the PARAGON-HF study, ARNI might exert a beneficial effect in women. Further, these findings suggest that ARNI may need to be more prescribed in women. We need further investigations to understand the reason for the underuse of GDMT in women and develop more appropriate treatment strategies for female HF patients.

## 6. Sex Differences in Clinical Outcome

A large number of clinical studies have been reported about the sex-related differences in the outcomes for HF patients. As shown in Table 2, conclusions for the sex-related differences in HF outcomes are inconsistent. This inconsistency of the sex-related differences in HF outcomes may result from a wide variety of patient characteristics in HF and different patient backgrounds in each study. In the majority of studies, the reported outcomes are similar between men and women or more favorable in women. However, several studies from Japan reported worse outcomes in women [74,100,101]. Particularly, sex-related differences were observed in the elderly [100], HFmrEF [74] or HFpEF [101]. Japan has the most advanced aging society, and the prevalence of elderly age, female sex, and HFpEF in HF patients has been dramatically rising [102,103,104]. The predominant female HFpEF population might be associated with worse outcomes in women because of limited effective treatment for HFpEF, highly prevalent comorbidities, and frailty in this population, As shown in our study [74], the lower implementation of GDMT in female HFmrEF might be associated with worse outcomes in this population. On the other hand, snEF was associated with worse outcomes compared to normal EF in women, which might be related to the pathophysiology of female snEF (see Section 4).

## 7. Future Perspectives

As mentioned above, female snLVEF and female HFrEF/HFmrEF patients have issues in improving their prognosis compared to male patients. Among female HFrEF/HFmrEF patients, more consideration for GDMT prescription might improve the prognosis of female HF patients. It may be necessary to reconsider the optimal medicine dose for males and females. Among female snLVEF patients, treatment for coronary microvascular dysfunction or sympathetic nerve overactivation has been suggested to improve their outcomes. One of the treatments for microvascular dysfunction and sympathetic nerve overactivation, substitution therapy for estrogen, which is expected to enhance the maintenance of autonomic nerve function, endothelial function, and coronary microcirculation, might be useful to improve the prognosis or prevent the development of the disease.

## 8. Conclusions

Substantial sex differences exist in heart morphology, function, and remodeling in terms of HF. Sex-related differences are also observed in GDMT prescription among HFrEF/HFmrEF patients and in the prognosis among snLVEF patients. We need further investigations in order to better understand the pathophysiology of female HFrEF/HFmrEF or snLVEF patients and improve their prognosis.

## Figures and Tables

**Table 1:** Previous reports on sex-related difference in GDMT implementation.

Authors	Number of Patients	HFrEF (%)	βB (%)	RASi (%) (ACEi/ARB/ARNI)		MRA (%)	SGLT2i (%)	Notes
Blumer et al., 2021 [75]	M: 3386 F: 1396	M: 100% F: 100%	M: 61.0, F: 56.0 *p* = 0.001	M: 63.4 F: 60.0 *p* = 0.028		M: 33.1 F: 30.8 *p* = 0.13	-	
Dewan P et al., 2019 [73]	M: 12,058 F: 3357	M: 100% F: 100%	M: 92.6, F: 91.6 *p* = 0.049	ACEi M: 88.7 F: 84.7 *p* < 0.001 ARB M: 11.9 F: 16.4 *p* < 0.001		M: 47.4 F: 46.3 *p* = 0.26	-	
Tromp et al., 2023 [76]	M: 6418 F: 2486	M: 100% F: 100%	M: 77 F: 75 *p* = 0.075	M: 68 F: 65 *p* = 0.236		M: 61 F: 56 *p* = 0.001	-	
Satake et al., 2014 [12]	M: 3234 F: 1502	(LVEF < 50%) M: 34.2 F: 24.9	M: 51.3 F: 43.9 *p* < 0.001	M: 78.6 F: 76.4 *p* = 0.101		-	-	
Lainscak et al., 2020 [77]	M: 6744 F: 2684	(LVEF ≤ 45%) M: 82.2 F: 63.3 *p* < 0.001	M: 90.2 F: 84.8 *p* < 0.001	M: 87.5 F: 80.6 *p* < 0.001		M: 59.8 F: 56.2 *p* = 0.001	-	
Gutierrez et al., 2020 [78]	M: 7454 F: 10062	unknown	M: 33.6 F: 30.8 *p* < 0.001	ACEi M: 33.1 F: 25.8 ARB M: 21.0 F: 23.8		M: 28.7 F: 23.7	-	
Yamamoto E et al., 2020 [79]	M: 2057 F: 1671	M: 45.1%, F: 27.5%	M: 70.2 F: 61.6 *p* < 0.0001	M: 60.7 F: 53.5 *p* < 0.0001		M: 44.5 F: 45.8 *p* = 0.43	-	
Daubert M A et al., 2021 [80]	M: 608 F: 286	M: 100% F: 100% (All patients LVEF ≤ 40%)	M: 94.7 F: 95.4 *p* = 0.74	M: 80.1 F: 78.1 *p* = 0.53		M: 50.5 F: 48.4 *p* = 0.57	-	
Cerlinskaite-Bajore K et al., 2021 [81]	M: 662 F: 416	M: 72.7% F: 60.1%	M: 37.6 F: 32.5 *p* = 0.089	M: 63.0 F: 66.0 *p* = 0.3102		M: 94.5 F: 95.2 *p* = 0.64	-	
Witting et al., 2023 [14]	M: 140,765 F: 3309	Mean LVEF = 31.7%	M: 73.3 F: 68.9 *p* < 0.001	RASi M: 76.4 F: 71.3 *p* < 0.001	ARNI M: 22.8 F: 21.5 *p* = 0.546	M: 27.8 F: 30.9 *p* = 0.002	-	
Pabon M et al., 2023 [82]	M: 6483 F: 1749	M: 100% F: 100% (All patients LVEF ≤ 35%)	M: 94.4 F: 93.8 *p* =0.28	ACEi M: 50.2 F: 45.3 *p* < 0.001 ARB M: 18.2 F: 23.6 *p* < 0.001	ARNI M: 20.0 F: 17.5 *p* = 0.020	M: 78.1 F: 76.4 *p* = 0.13	M: 2.9 F: 1.5 *p* < 0.001	
Russo G et al., 2021 [83]	M: 441 F: 167	M: 100% F: 100%	M: 69.4 F: 70.2	M: 83.7 F: 83.3		M: 40.4 F: 38.1		
Kawai et al., 2023 [74]	M: 2357 F: 1586	(LVEF < 40%) M: 48% F: 29% (40% ≤ LVEF < 50%) M: 18% F: 17%	(LVEF < 40%) M: 87 F: 86 *p* = 0.84 (40% ≤ LVEF < 50%) M: 83 F: 75 *p* = 0.022	(LVEF < 40%) M: 69 F: 68 *p* = 0.95 (40% ≤ LVEF < 50%) ACEi/ARB M: 66 F: 60 *p* = 0.088		(LVEF < 40%) M: 44 F: 46 *p* = 0.49 (40% ≤ LVEF < 50%) M:29 F:36 *p* = 0.099		(LVEF < 40%) RASi + βB M: 62, F: 62 *p* = 0.78 RASi + βB + MRA M: 30, F: 31 *p* = 0.68 (40% ≤ LVEF < 50%) RASi + βB M: 56, F: 44 *p* = 0.002 RASi + βB + MRA M: 18, F: 18 *p* = 0.96

HFrEF, heart failure with reduced ejection fraction; RASi, renin–angiotensin system inhibitor; ACEi, angiotensin-converting enzyme inhibitor; ARB, angiotensin receptor blocker; ARNI, angiotensin receptor neprilysin inhibitor; βB, β blocker; MRA, mineralocorticoid receptor antagonist; SGLT2i, sodium-glucose cotransporter 2 inhibitor; M, male; F, female; LVEF, left ventricular ejection fraction.

**Table 2:** Previous studies exploring the sex-related differences in clinical outcomes in real-world HF population.

	Authors	Region	Number of Patients	LVEF	Outcomes of Women (Compared with Men)	Notes
All LVEF	Akcay F et al., 2023 [105]	Turkey	M: 918 F: 688	All	In-hospital mortality rate ↑	
	Muhammed T et al., 2021 [106]	Dutch	M: 14,517 F: 11,259	All	HF hospitalization ↓ All-cause death ↓	
	Nozaki A et al., 2017 [100]	Japan	M: 696 F: 354	All	All-cause death ↑ (Age ≥ 79: ↑, Age < 79: →)	
	Yamamoto E et al., 2020 [79]	Japan	M: 2057 F: 1671	All	HF hospitalization → All-cause death →	The percentage of HFrEF patients M: 45.1%, F: 27.5%
	Basic et al., 2022 [107]	Sweden	M:2781 F:971	All	all-cause mortality →	Age: 18–54 years
	Kim et al., 2023 [108]	Korea	M: 2993 F: 2632	All	In-hospital mortality ↓ CV death ↓ All-cause death ↓ All-cause death + HF rehospitalization ↓	
	Cenko E et al., 2019 [109]	European 12 countries	M: 7331 F: 3112	All	30-days mortality ↑ Killip class ≥ II ↑	After STEMI treatment
	Kawai et al., 2023 [74]	Japan	M: 2357 F: 1586	All	Cardiac death + HF rehospitalization →	
HFrEF	Dewan et al., 2019 [73]	Worldwide	M: 12,058 F: 3357	LVEF ≤ 40%	HF hospitalization ↓, CV death ↓ All-cause death ↓ KCCQ score ↓	
	Russo G et al., 2021 [83]	Italy	M: 441 F: 167	LVEF < 40%	All-cause death → HF progression ↓	
	Kawai et al., 2023 [74]	Japan	M: 1142 F: 462	LVEF < 40%	Cardiac death + HF rehospitalization →	
HFmrEF	Russo G et al., 2021 [83]	Italy	M: 300 F: 135	LVEF 40–49%	All-cause death → HF progression →	
	Kawai et al., 2023 [74]	Japan	M: 434 F: 273	LVEF 40–49%	Cardiac death + HF rehospitalization ↑	
HFpEF	Lam C S.P. et al., 2012 [110]	Worldwide	M: 1637 F: 2491	LVEF ≥ 45%	All cause death ↓ All cause hospitalization + death ↓	Age ≥ 65
	Zsilinszka et al., 2016 [111]	USA	M: 1353 F: 2808	LVEF ≥ 40%	180 day all cause death → Hospitalization due to any cause →	
	Dewan P et al., 2019 [112]	Worldwide	M: 4010 F: 4458	LVEF ≥ 45%	HF hospitalization → All-cause death ↓ KCCQ ↓	
	Sotomi Y et al., 2021 [101]	Japan	M: 389 F: 481	LVEF > 50%	All-cause death + HF hospitalization ↑	
	Kawai et al., 2023 [74]	Japan	M: 781 F: 851	LVEF > 50%	Cardiac death + HF rehospitalization →	snEF associated with worse outcome compared to normal EF in women

CV, cardiovascular; M, male; F, female; HF, heart failure; HFrEF, heart failure with reduced ejection fraction; HFmrEF, heart failure with mildly reduced ejection fraction; HFpEF, heart failure with preserved ejection fraction; KCCQ, Kansas City Cardiomyopathy Questionnaire; LVEF, left ventricular ejection fraction; STEMI ST-elevated myocardial infarction.

## Data Availability

This study did not report any data.
